# Wearable Fall Detector using Integrated Sensors and Energy Devices

**DOI:** 10.1038/srep17081

**Published:** 2015-11-24

**Authors:** Sungmook Jung, Seungki Hong, Jaemin Kim, Sangkyu Lee, Taeghwan Hyeon, Minbaek Lee, Dae-Hyeong Kim

**Affiliations:** 1 Center for Nanoparticle Research, Institute for Basic Science (IBS), Seoul, 151-742, Republic of Korea; 2 School of Chemical and Biological Engineering, Institute of Chemical Processes, Seoul National University, Seoul, 151-742, Republic of Korea; 3 Department of Physics, Inha University, Incheon, 402-751, Republic of Korea

## Abstract

Wearable devices have attracted great attentions as next-generation electronic devices. For the comfortable, portable, and easy-to-use system platform in wearable electronics, a key requirement is to replace conventional bulky and rigid energy devices into thin and deformable ones accompanying the capability of long-term energy supply. Here, we demonstrate a wearable fall detection system composed of a wristband-type deformable triboelectric generator and lithium ion battery in conjunction with integrated sensors, controllers, and wireless units. A stretchable conductive nylon is used as electrodes of the triboelectric generator and the interconnection between battery cells. Ethoxylated polyethylenimine, coated on the surface of the conductive nylon electrode, tunes the work function of a triboelectric generator and maximizes its performance. The electrical energy harvested from the triboelectric generator through human body motions continuously recharges the stretchable battery and prolongs hours of its use. The integrated energy supply system runs the 3-axis accelerometer and related electronics that record human body motions and send the data wirelessly. Upon the unexpected fall occurring, a custom-made software discriminates the fall signal and an emergency alert is immediately sent to an external mobile device. This wearable fall detection system would provide new opportunities in the mobile electronics and wearable healthcare.

Falls often cause severe health consequences especially in the aged with the possible injuries including bone fractures or intraperitoneal and intracranial bleedings. The occurrence of such states without consciousness is particularly fatal. This necessitates means of detecting a fall and immediately sending an alarm to the nearest emergency clinic through wireless communications. However, the conventional automatic fall detection system is rigid and bulky due in large to its use of heavy power supply systems and often has power management problems^1^. These features impede the mobility of the user during the daily life and create much discomfort.

Wearable devices have received much attention recently. Numerous innovations have been reported, such as wearable human-machine interfaces^2^,^3^,^4^,^5^, skin-based biomedical/electronic devices^6^,^7^,^8^,^9^,^10^,^11^,^12^,^13^, wearable displays^14^, and highly sensitive sensors for artificial skins^15^,^16^,^17^, accompanied with intensive mechanics studies^18^,^19^. The combination of flexible/stretchable devices with commercial microchips^20^,^21^ improves performances. These achievements have enabled a shift from rigid, bulky, and heavy electronic devices to stretchable, thin, and light-weight ones. However, the application of these breakthroughs in existing fall detection electronic systems, particularly in energy harvesting and storage modules of the integrated platform, remains challenging.

Various types of deformable supercapacitors and batteries have been recently reported. These have used origami techniques^22^,^23^, serpentine/wavy structures^24^,^25^, strain isolating islands^26^,^27^, or buckled electrodes^28^,^29^. Intrinsically flexible and stretchable materials were also used as device components^30^,^31^,^32^,^33^. In addition, power generation units, such as piezo- and tribo-electric generators (PEGs and TEGs) converting human body movements into the electrical energy, have been in high interests^34^,^35^,^36^,^37^. These were used to recharge the energy storage modules^38^,^39^,^40^,^41^ and/or slow down their discharge. PEGs and TEGs were also used to measure physical properties^42^,^43^,^44^,^45^,^46^,^47^,^48^,^49^ and directly turn on the light-emitting diodes^50^,^51^,^52^,^53^,^54^. Furthermore, various strategies to enhance the power generation efficiency of the TEGs have been recently reported^55^,^56^.

However, with all these accomplishments, high performance stretchable energy harvesting (TEGs) and storage (lithium ion batteries; LIBs) devices are not yet seamlessly integrated with sensors/controllers and wireless communication units for the wearable fall detection system. More optimizations and performance improvements in stretchable TEGs, LIBs, and their integrated systems are also required. Toward addressing these issues, we propose a wearable fall detector that uses a wristband-type TEG and LIB. In TEG, its surface energy state is engineered to enhance its electrical performance. Such manipulation in TEG changes surface properties like the hydrophobicity and triboelectricity, however preserves the mechanical behavior including stretchability. The LIB shows the high stretchability while maintaining stable electrochemical performances. The TEG and LIB are interconnected to the microcontroller (MCU) through conductive carbon fibers and supply power to the fall detection system. The integrated system successfully detects a fall from normal daily motions and sends an immediate alarm wirelessly.

## Results

### Overview of the wearable fall detection system

**Figure 1:**
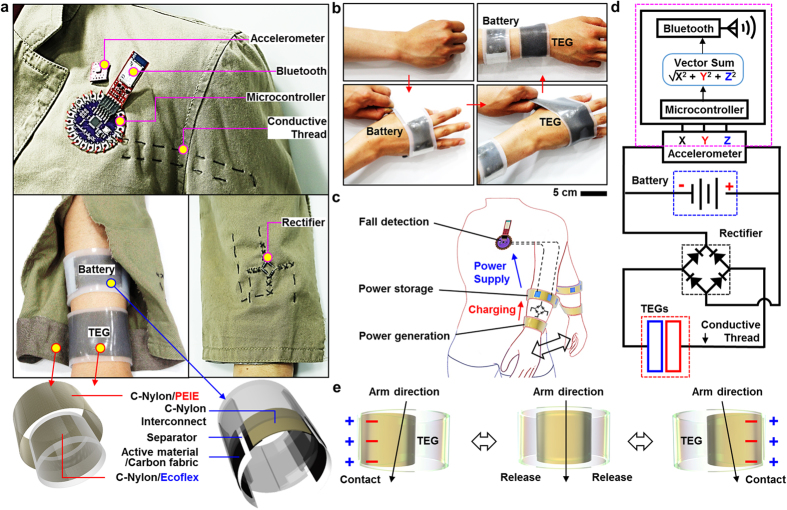
Images and schematic illustrations of the wearable fall detection system. (**a**) Fall detecting sensor and related electronics attached onto a jacket (top), wristband-type energy devices worn on the body (middle left), rectifier sewn on the jacket (middle right), and corresponding schematic illustrations of individual energy devices (bottom). (**b**) Sequence of wearing the stretchable TEG and LIB. (**c**) Illustration of overall operation sequence of the fall detection system. (**d**) Circuit diagrams of the wearable fall detection system. (**e**) Schematic summary of the energy harvesting process of the wristband-type stretchable TEG.

Figure 1 presents an overview of the material strategies, individual devices, integrated system designs, and operation mechanisms of the proposed wearable fall detector. The system is composed of stretchable power supply devices (TEG and LIB) that are interconnected with a MCU for data processing, a three-axis accelerometer for fall detection, and a Bluetooth module for data transmission. The stretchable power supply devices are connected to the electronics mounted on a jacket through conductive carbon threads (Fig. 1a) and are worn on the wrist (Fig. 1b). The other devices including the accelerometer and the MCU interconnected with Bluetooth module are sewn to clothing in the chest region. The power generated by the TEG by movements of the arm while walking or running is transmitted via conductive threads to rectifiers and then the LIB. The charged LIB supplies power to the fall detection sensor and electronics. A schematic of the system and a circuit diagram are shown in Fig. 1c,d, respectively.

**Figure 2:**
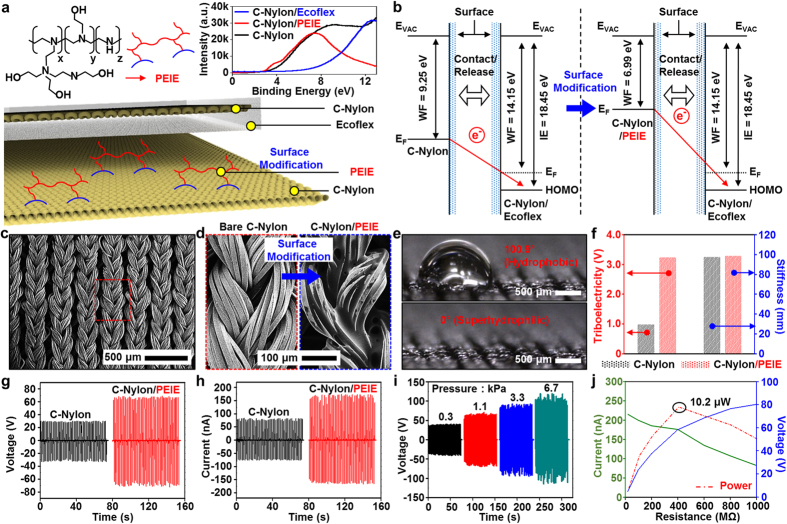
Work function control and related materials strategy of the wearable TEG. (**a**) Chemical structure of the surface modifier (PEIE) (left top); photoemission cutoff of C-Nylon, C-Nylon/PEIE, and C-Nylon/Ecoflex, measured by UPS (right top); and schematic illustration of the TEG (bottom). (**b**) Mechanism of contact electrification between the bare C-Nylon and Ecoflex coated C-Nylon (left); the PEIE-coated C-nylon and Ecoflex-coated C-Nylon (right). **(c)** Low-magnification SEM image of bare C-Nylon. (**d**) Enlarged SEM images for comparison of the morphological difference between the bare C-Nylon (left) and PEIE-coated C-Nylon (right). (**e**) Images of a water droplet on the bare C-Nylon (top) and PEIE-coated C-Nylon (bottom) for determination of the difference between the surface energies of two materials. (**f**) Changes in the electrical (left; triboelectricity) and mechanical (right; stiffness) properties of C-Nylon by PEIE modification. (**g**) Open-circuit voltage and (**h**) short-circuit current induced by contact/release of the TEG with the bare C-Nylon (black) and C-Nylon/PEIE (red). (**i**) Open-circuit voltage generated by the TEG under different contact pressures (0.3, 1.1, 3.3, and 6.7 kPa, respectively). (**j**) Short circuit current (green) and open circuit voltage (blue) depending on the external resistance. The calculated output power (red dotted line) shows that the maximum output power is achieved when R = 400 MΩ.

**Figure 3:**
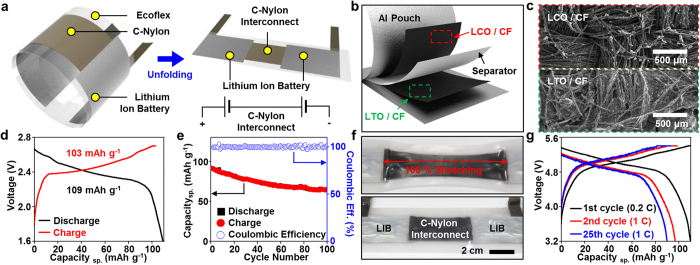
Overview of the wristband-type stretchable LIB. Schematic illustration of (**a**) a wristband-type LIB and (**b**) the single pouch cell (exploded view). (**c**) SEM images of LCO (top) and LTO (bottom) coated on carbon fabric electrodes. (**d**) 1st charge-discharge curve of a full cell, and (**e**) its cycle performance and coulombic efficiency. (**f**) Pictures of wristband-type LIB before (bottom) and after ~100% stretching (top). (**g**) Charge-discharge curve of the LIB with repetitive stretching/releasing (cycling speed: 60 rpm; applied strain: 50%) at the 1st cycle (current density: 0.2 C; black), 2nd cycle (current density: 1 C; red), and 25th cycle (current density: 1 C; blue).

**Figure 4:**
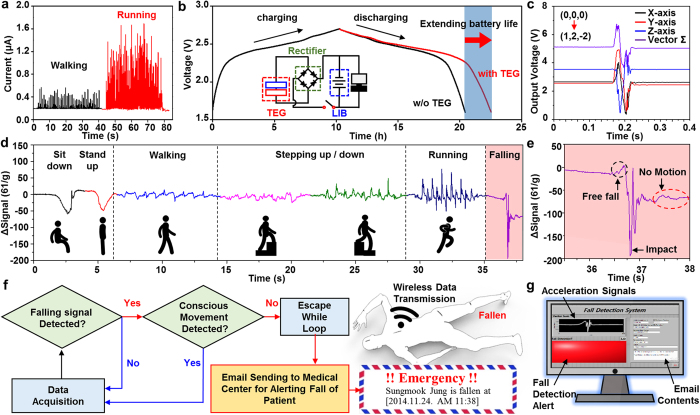
Demonstration of the fall detector powered by the stretchable TEG and LIB. (**a**) Electricity generated by the TEG from daily living activities (walking and running) (**b**) Comparisons of discharging characteristics of the LIB with (red line)/without (black line) co-operation of the TEG. (**c**) Output voltage changes of the three-axis accelerometer during sudden movement from (0,0,0) to (1,2,-2) and their vector sum (black: x-axis, red: y-axis, blue: z-axis, purple: vector sum). (**d**) Temporal changes of the vector sum during various movements of the subject. (**e**) Enlarged view of the vector sum during a fall. (**f**) Flow chart of the fall detection system, which includes an algorithm for monitoring unexpected falls and sending an alert email when a fall is detected. Inset illustrates the fall accident. (**g**) Front panel of the fall detection program, which includes a graphic indicator of the wirelessly transmitted signal of the accelerometer, an LED that notifies a fall, and a user interface.

The wearable TEG comprises two different material components facing each other. The first is an ethoxylated polyethylenimine (PEIE)-coated conductive nylon (C-Nylon) and the other is a silicone (Ecoflex®)-coated C-Nylon. This stretchable TEG is capable of harvesting the electrical energy from daily human movements such as the swinging of arms. The movement brings the two material components into contact, resulting in the generation of friction. Subsequent breakage of the contact is accompanied by the generation of positive and negative charges on different surfaces due to the contact electrification (Fig. 1e and Supplementary Fig. S1). Figure 2 provides more details on the TEG. The wearable wristband-type LIB consists of carbon fabrics coated with active materials for the cathode and anode, a polypropylene separator, and stretchable C-Nylon interconnect (Fig. 1a bottom). Details of the wristband-type LIB are presented in Fig. 3. The output voltage generated by the accelerometer when accelerated during a fall is transmitted to the MCU. A customized software embedded in the MCU calculates the vector sum of the three-axis acceleration (x, y, and z) and sends the data and an alarm to external devices via a Bluetooth module (see Fig. 4 and Supplementary Movies 1 for details).

### Work function control and performances of the wearable triboelectric generator

The amount of charges generated by the TEG is generally proportional to the work function (WF) difference between two material components contacted each other (Supplementary Fig. S2)^57^,^58^. The WF of materials utilized in the TEG is uniquely engineered for the superior charge generation in the process of the contact electrification (Fig. 2). A silicone-based rubber (Ecoflex) and PEIE are selected for large and small WF materials in surface modifications, respectively. A material with large WF tends to attract electrons from that of small WF and holds it on the surface. The C-Nylon is utilized as a substrate owing to its high stretchability and conductivity. Each WF control material is coated on the top surface of C-Nylons and physical property examinations of modified C-Nylons are conducted. Besides Ecoflex as an electron acceptor in the TEG, another silicone-based polymer, polydimethylsiloxane (PDMS), is also investigated for the comparison (Supplementary Fig. S3). As electron acceptors in a triboelectric generator, the C-Nylon coated with Ecoflex (WF = 14.07 eV) shows the superior attraction of electrons (Supplementary Fig. S3a,b) over that coated with PDMS (WF = 13.2 eV). Furthermore, the low Young’s modulus of Ecoflex improves the stretchability and softness (Supplementary Fig. S4), which helps conformal contacts between two components of the TEG. For the WF control of the other component such as an electron donor in the TEG, PEIE was utilized as a surface modifier. The dipole moments of the neutral amine groups of PEIE (Fig. 2a, top left) and the charge transfer characteristics of the material reduce its WF^59^, thereby enhances the transfer of electrons to the other part of the TEG. The use of up to six coatings of PEIE (20 wt%, Supplementary Fig. S5) modifies a WF from 9.25 to 6.99 eV (modified band diagram in Fig. 2b), as measured by ultraviolet photoemission spectroscopy (UPS) (Fig. 2a top right and Supplementary Fig. S3c). The control of WFs maximizes the charge separation in the contact electrification process and therefore leads to the higher performance in the electrostatic power generation.

Owing to the twisted mesh structure of the soft fabric and elastomeric and ultrathin polymeric coatings (see scanning electron microscope (SEM) images in Fig. 2c,d), the modified C-Nylons are stretchable up to 125% without mechanical damages (Fig. 1b and Supplementary Fig. S4). The PEIE treatment changes the morphological (Fig. 2d), chemical (Fig. 2e), and triboelectric (Fig. 2f, left red axis) properties, but does not affect the original mechanical properties (Fig. 2f, right blue axis). The surface property change from hydrophobic to superhydrophilic (Fig. 2e) confirms the complete modification of the surface with PEIE molecules. The triboelectric voltage of the C-Nylon was increased to three-fold (Fig. 2f, left) by these WF controls. The generated triboelectricity and mechanical property of the material were measured by following the standard procedures of Korean triboelectric voltage measurement and Korean heart loop test, respectively.

The improved triboelectric power generation owing to the surface treatment is shown in Fig. 2g,h. After the WF engineering via PEIE, open-circuit voltage *V*_*OC*_ and short-circuit current *I*_*SC*_ increase from 29 to 66 V and from 78 to 159 nA, respectively. As the contact pressure increases, the TEG performance (voltage) proportionally increases from 36 V (under 0.3 kPa) to 104 V (under 6.7 kPa) (Fig. 2i). For all other experiments, the fixed contact pressure and frequency of 1.1 kPa and 0.7 Hz were used. These values correspond to the pressure and frequency condition of contacts between the sleeve and wrist during the normal daily activity. The thickness of Ecoflex coating and its load resistance were also fixed at 2 mm and 400 MΩ, respectively, which correspond to the optimum power generation condition (Fig. 2j). As shown in Supplementary Fig. S6a and b, the rectified voltage and current of ~75 V and ~190 nA are obtained. The corresponding accumulated power and charge are also shown in the right axis of Supplementary Fig. S6a and b, respectively.

### Wristband-type lithium ion battery

Figure 3a shows schematic illustrations describing components and their connection layout of the wristband-type LIB. Two flexible battery cells interconnected by the stretchable C-Nylon constitute the LIB that powers the wearable fall detection system. The C-Nylon interconnect gives appropriate stretchability to the wearable battery as well as electrically connects two cells in series. The series connection meets the voltage requirement for the operation of the MCU (2.7–5.5 V). The voltage regulator on the MCU supplies regulated operation voltage to the accelerometer. Note that the aluminum pouch and the flexible carbon fabric (Fig. 3b and Supplementary Fig. S7a) are used as the encapsulation and current collectors, respectively. The flexible/stretchable LIB makes conformal contacts with the human wrist (Fig. 1b)^35^,^36^. Cyclic voltammetry (Supplementary Fig. S7b) exhibits an excellent electrochemical stability of the carbon fabric as a current collector in the potential range of 1.0–4.2 V (*vs* Li/Li^+^), which corresponds to the operating voltage (Supplementary Fig. S7c) of lithium cobalt oxide (LCO) and lithium titanate (LTO). These active materials (LCO and LTO) are well coated on the surface of carbon fabric (Fig. 3c).

The electrochemical performances of the LCO and LTO electrodes are evaluated by a galvanostatic charge/discharge test using coin cells (CR2032). Supplementary Fig. S7c shows the charge-discharge curves for half cells at a current density of 0.2 C (1 C = 140 mA g^−1^ for LCO and 175 mA g^−1^ for LTO). Specific capacities of LCO and LTO half cells are 127 and 171 mAh g^−1^, respectively. Each half cell maintains its initial capacity without any capacity decrease up to 100 cycles at a current density of 1 C (Supplementary Fig. S7d). Using these electrodes, a full cell was assembled in a flexible pouch and its electrochemical performance was evaluated. At a current density of 0.2 C, the full cell shows the discharge capacity of 109 mAh g^−1^ (Fig. 3d). After 100 cycles at a current density of 1 C, it still delivers 70.5% of the initial capacity, indicating stable charging and discharging characteristics (Fig. 3e). The coulombic efficiency of the cell is >99% after 100 cycles.

C-Nylon has excellent stretchability with a negligible variation of the electrical conductivity under a strain of 100%^30^. Due to the elastic nature of the C-Nylon interconnect and Ecoflex, the LIB can be stretched up to ~100% (Fig. 3f). The Ecoflex encapsulation protects junctions between the C-Nylon and battery cells under applied strains (Supplementary Fig. S7e and f). Figure 3g shows charge-discharge curves of the LIB while stretched/released under ~50% applied strain (typical strain induced in wearing wristbands) at a cycling rate of 60 rpm. At the first charge-discharge cycle (current density: 0.2 C), no change in the capacity and voltage was observed in comparison with the original electrochemical performance (Supplementary Fig. S7g). Then the current density was increased to 1 C for multiple cycles (>25 times) while the LIB was continuously stretched and released. The results show the stable cycle performance comparable to the original single cell data evaluated without stretching (Fig. 3e). The stretchable C-Nylon interconnect relieves the induced strain and thereby the external strain is not directly applied on the wristband-type LIB cells and the stable electrochemical performances are obtained.

## Discussion

Figure 4 illustrates the monitoring of a series of simulated motions during daily life by using the integrated wearable fall detector (see Supplementary Movie 1). Movements with the higher impact and/or frequency (e.g., running versus walking) increase the power generation of the TEG (Fig. 4a). In this work, we utilized a TEG for prolonging the discharge time of a LIB rather than charging a LIB. Previously, we have demonstrated integrated energy devices for a wearable activity monitor, which were composed of fabric-based TEGs and supercapacitors^32^. In the case using supercapacitors (SC), TEGs can fully charge SCs. However, the total energy stored in the SCs was mostly not enough for long-time operation of electronic devices. Meanwhile, a LIB utilized in this system has the high energy density enabling long-term operation of devices including the fall detector. Considering the power output of a TEG and the energy density of a LIB, we utilized TEGs as a power extending unit rather than a designated charger. The fall detector exhibited long-lasting performance with the TEG.

Figure 4b shows the voltage profile of the LIB interconnected with TEG. LIB was charged with an applied current of ~21 μA without the TEG. The continuous recharging of the battery by the TEG prolongs the battery life from 10 h to 12 h 30 min at the same applied current of 21 μA. The contact and release of the TEG was conducted using a customized bending stage operating in the speed of 4 Hz with the displacement of ~2 cm. Considering the operation current of the integrated accelerometer (700 μA), it can be estimated that the TEG prolongs the use time of the sensor as prolonged operation time/charging time = 0.45 sec/min. The slower power consumption and faster movements elongate hours of use further. The accelerometer powered by the LIB distinctively responses to the direction and the amount of movements (Fig. 4c).

Supplementary Movie 1 demonstrates the integrated operation of energy devices with sensors and electronics. The detailed vector sum of the sensor (accelerometer) for different motions is shown in Fig. 4d. As the motion becomes more active, the signal intensity increases. A fall produces the largest peak-to-peak signal amplitude. Repeated measurements show similar reproducible patterns in the same specific motion but with discriminated ones between different motions (Supplementary Fig. S8). The data are processed in the MCU for *in situ* wireless transmission to external devices via the Bluetooth module. The fall produces a distinctive signal, i.e. a slight increase of the signal due to the free fall right before the impact, a large impact signal upon the hit to the ground, and a flat signal in the motionless state on the ground (Fig. 4e). The custom-made algorithm (Fig. 4f) and LabVIEW-based program (Supplementary Fig. S9) recognize the occurrence of a fall by detecting these characteristic patterns. An email alert is sent to the emergency medical facility automatically (Fig. 4g and Supplementary Movie 1). An emergency alarm is not triggered by any motions except for the fall.

In summary, the wearable fall detection system is presented, integrating the stretchable TEG and LIB with fall detection electronics. The energy generation devices (TEGs) use a stretchable conductive substrate (C-Nylon) whose work function is modified for improving the triboelectric generation performance. The energy storage devices (LIBs) also use the same substrate as interconnection to enhance stretchability and wearability. The different device modules are interconnected by sewing onto a jacket using conductive threads. The stretchable and thin components afford softness, wearability, and light weight, hence accomplishing a truly mobile system. This system would contribute to prevent fatal events from unexpected falls particularly for the elderly.

## Methods

### Fabrication of the wristband-type stretchable TEG

To fabricate the TEG, C-Nylon (Meditex 130, Sparkfun, Germany) was coated with PEIE (80% ethoxylated solution, Sigma-Aldrich, USA) diluted in ethanol (99.9% anhydrous, Samchun, Korea) with a concentration of 4–20 wt%. The solution was casted on the C-Nylon and dried in a convection oven at 55 °C for 6 h. The process of casting and drying was repeated six times. The PEIE-coated fabric was then tailored and sewn onto clothing using conductive carbon threads. To fabricate the other side of the TEG, C-Nylon was coated with Ecoflex (0030 Ecoflex®, Smooth-On, USA). Ecoflex elastomer was prepared by mixing its prepolymer and crosslinker at a weight ratio of 1:1. After coating, it was cured in the convection oven at 70 °C for 4 h.

### Fabrication of the wristband-type stretchable LIB

To prepare electrodes for the LIB, active materials, carbon black, and polyvinylidene difluoride (PVdF, Kynar 2801) were mixed at a weight ratio of 7:2:1. LCO (Sigma Aldrich, USA) and LTO (Sigma Aldrich, USA) powders were used as active materials for cathode and anode respectively. Carbon fabrics were coated with each slurry and then dried in a vacuum oven at 70 ^o^C for >12 h. The areal mass loading of each active material was controlled to be 3 mg cm^−2^. Coin half cells (CR2032) were prepared by assembling each working electrode, a lithium foil (used as reference and counter electrodes), and a polypropylene separator. The electrolyte of 1M LiPF_6_ dissolved in a mixture of ethylene carbonate (EC) and diethylene carbonate (DEC) (v/v = 1:1, Panax Etec Co., Korea) was used. The full cell was assembled by sandwiching a separator between a cathode (1.7 × 3.8 cm^2^) and an anode (2 × 4 cm^2^) and then sealed in a flexible aluminum pouch. Finally, the same electrolyte was injected into the pouch cell. For the wristband-type LIB, two pouch cells were connected in series by C-Nylon interconnect using a conductive thread and then fully covered with the Ecoflex. The Ecoflex was cured at room temperature over 1 h. For charging-discharging tests of LIB with TEG, a pouch cell was fabricated using cathode and anode with the area of 1 × 1 and 1.2 × 1.2 cm^2^, respectively.

### Acquisition of the vector sum from the accelerometer

Human motions were detected by a three-axis accelerometer (LIS344ALH, STMicroelectronics, USA). The accelerometer has ±2 g (where g is gravitational acceleration) measurement range. It consumes the low power (~700 μA) and is operated in the voltage range of 2.4–3.6 V. The accelerometer was powered by the LIB, which was charged by the co-integrated TEG. The voltage changes that reflect the acceleration along the x-, y-, and z-axis were recorded by the MCU (Arduino pro mini, Sparkfun, USA). The recorded data were analyzed and the vector sum was calculated by the custom-made software embedded in the MCU. The data were simultaneously transferred to the external device via the Bluetooth module (BlueSMiRF HID, Sparkfun). The custom-made LabVIEW-based program plotted the transferred signals, saved the received data for post-processing, decided whether the user was fallen or not, and sent an email to the predetermined address if a fall was detected.

### Optical, physical, and electrical characterizations

The morphologies of the samples were characterized by a digital single-lens reflex camera (Cannon EOS 600D, Cannon, Japan) and SEM (S-3400N, Hitachi, Japan). The WF of the C-Nylon, C-Nylon/PEIE, C-Nylon/Ecoflex, and C-Nylon/PDMS were measured by x-ray photoelectron spectroscopy (Theta probe, Thermo Fisher Scientific Co.). The triboelectricity was measured using the standard Korean standard test procedure (KSK 0555) and the stiffness was measured by heart loop test procedure (KSK 0538). The electrochemical measurements were done using a potentiostat (600E Potentiostat/Galvanostat, CH Instruments, USA). The electrical current and voltage output were monitored by the parameter analyzer (B1500A, Agilent, USA).

### Ethical approval and informed consent for experiments involving human subjects

Experiments are conducted according to the protocols and guidelines approved by animal care committee at Seoul National University. In addition, an informed consent is acquired from the participant before conducting the experiments.

## Additional Information

**How to cite this article**: Jung, S. *et al.* Wearable Fall Detector using Integrated Sensors and Energy Devices. *Sci. Rep.* **5**, 17081; doi: 10.1038/srep17081 (2015).

## Supplementary Material

Supplementary Information

Supplementary Movie S1
