# Supplementary material for: Wearable Fall Detector using Integrated Sensors and Energy Devices
Source: Sci Rep. 2015 Nov 24;5:17081. doi: 10.1038/srep17081 (PMC4657018; doi:10.1038/srep17081)
Supplement: Supplementary Information [file srep17081-s1.pdf]

Supplementary Information

Wearable Fall Detector using Integrated Sensors and Energy Devices

Sungmook Jung[1,2,†], Seungki Hong[1,2,†], Jaemin Kim[1,2,†], Sangkyu Lee[1], Taeghwan Hyeon[1,2], Minbaek Lee[3], and Dae-Hyeong Kim[1,2,*]

[1]*Center for Nanoparticle Research, Institute for Basic Science (IBS), Seoul, 151-742, Republic of Korea*

[2]*School of Chemical and Biological Engineering, Institute of Chemical Processes, Seoul National University, Seoul, 151-742, Republic of Korea*

[3]*Department of Physics, Inha University, Incheon, 402-751, Republic of Korea*

†*These authors contributed equally to this work.*

*To whom correspondence should be addressed.

E-mail: dkim98@snu.ac.kr

Supplementary 1

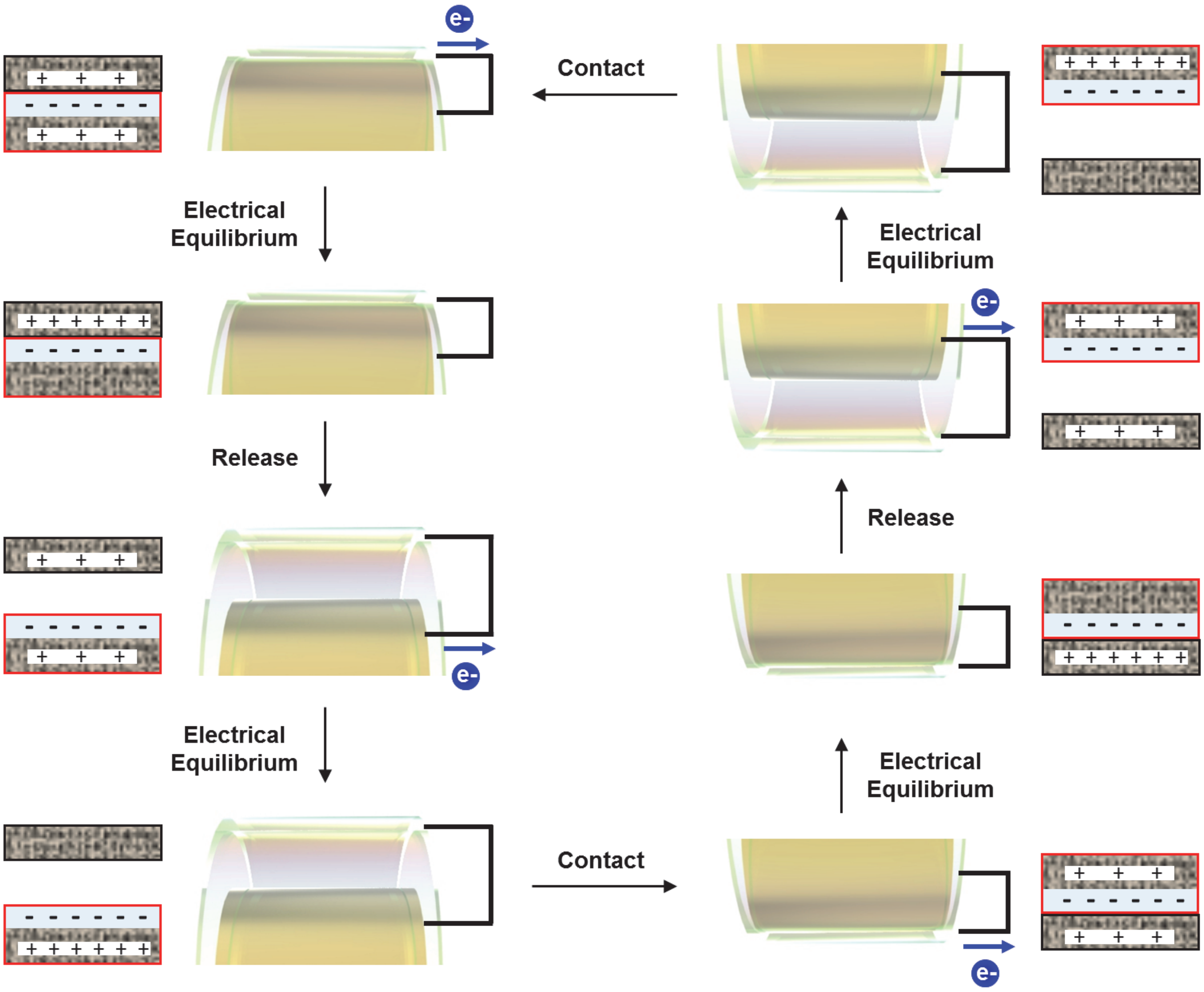

Figure S1 | Schematic diagram showing the process by which electricity is generated through contact/release.

Supplementary 2:

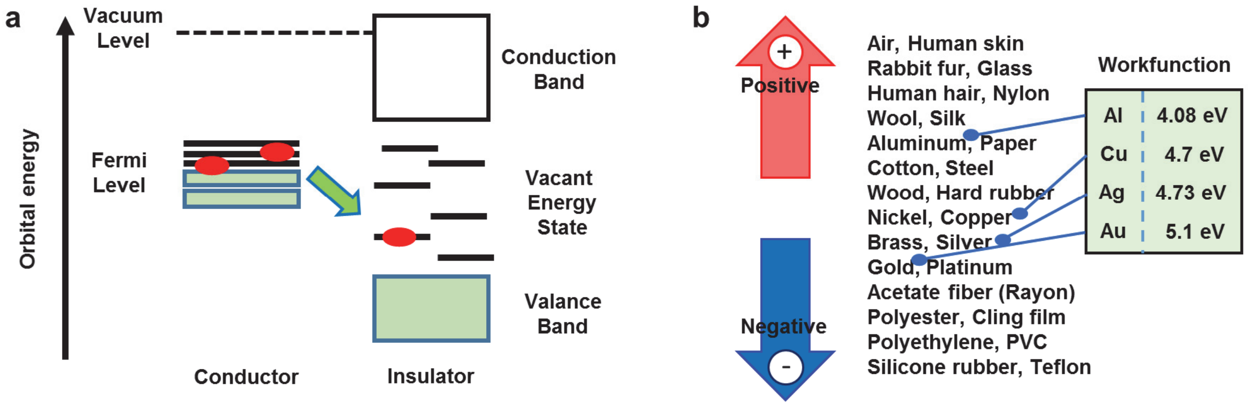

Figure S2 | (a) Charge produced by contact between conductor and insulator. **(b)** Triboelectric series (left) and work function (WF) series (right). Contact electrification, also known as triboelectric charging, occurs when two different materials make contact. The surface of the material that has a stronger electron affinity will be negatively charged after separation, while the surface of the other will contain an equal amount of positive charges. Figure S1a briefly illustrates the mechanism of triboelectric charging.[1,2] The WF significantly affects the polarity. As shown in Figure S1b, the polarity is empirically described in the form of a triboelectric series, the order of which matches well with the order of the WF in metals. The triboelectric series can be expanded to insulators with electron affinity or molecular ionic state.[3] However, there are many other factors that determine the polarity, such as the electronic properties, state density, hydrophilicity, surface roughness, surface stress/strain, and impurities. When a material that is relatively negative on the triboelectric series makes contact with another material that is relatively positive, electrons would move from the latter to the former.[4] In this study, silicone rubber (Ecoflex) was used as the negative material and PEIE-coated C-Nylon as the positive material. It should be noted that the function of the PEIE was to reduce the WF of the C-Nylon, thereby increasing the difference of WFs between the materials of the TEG, and consequently enhancing the electric power generation.

Supplementary 3:

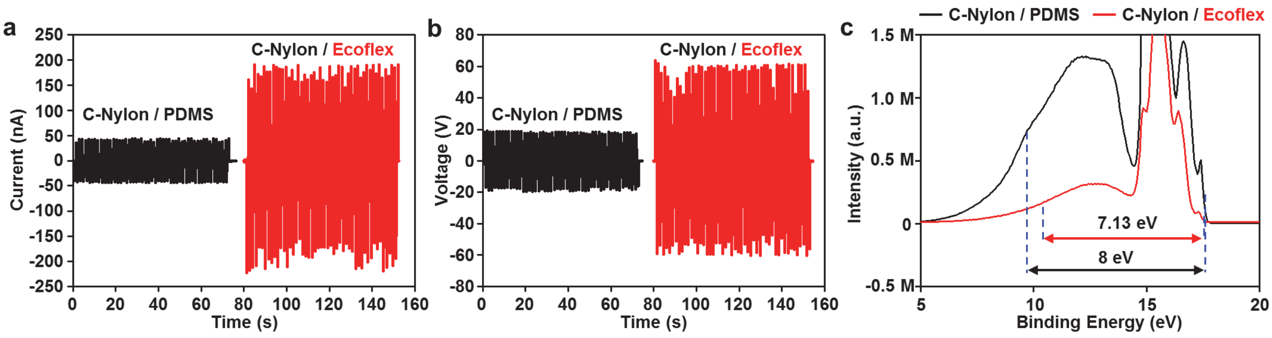

Figure S3 | Comparison of electric power generation using C-Nylon/PDMS and C-Nylon/Ecoflex. **(a)** Short-circuit current. **(b)** Open-circuit voltage. **(c)** UPS spectrum.

Supplementary 4:

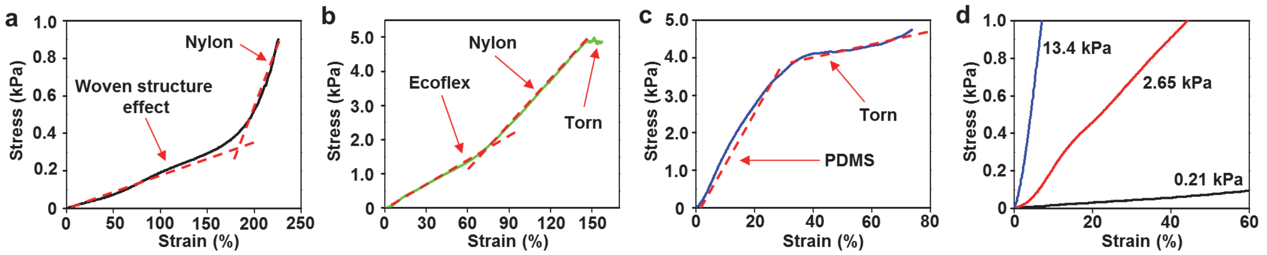

Figure S4 | Comparison of the mechanical properties of bare C-Nylon, C-Nylon/Ecoflex, and C-Nylon/PDMS. Stress–strain curves of **(a)** bare C-Nylon, **(b)** C-Nylon/Ecoflex, and **(c)** C-Nylon/PDMS. **(d)** Young's modulus.

Supplementary 5:

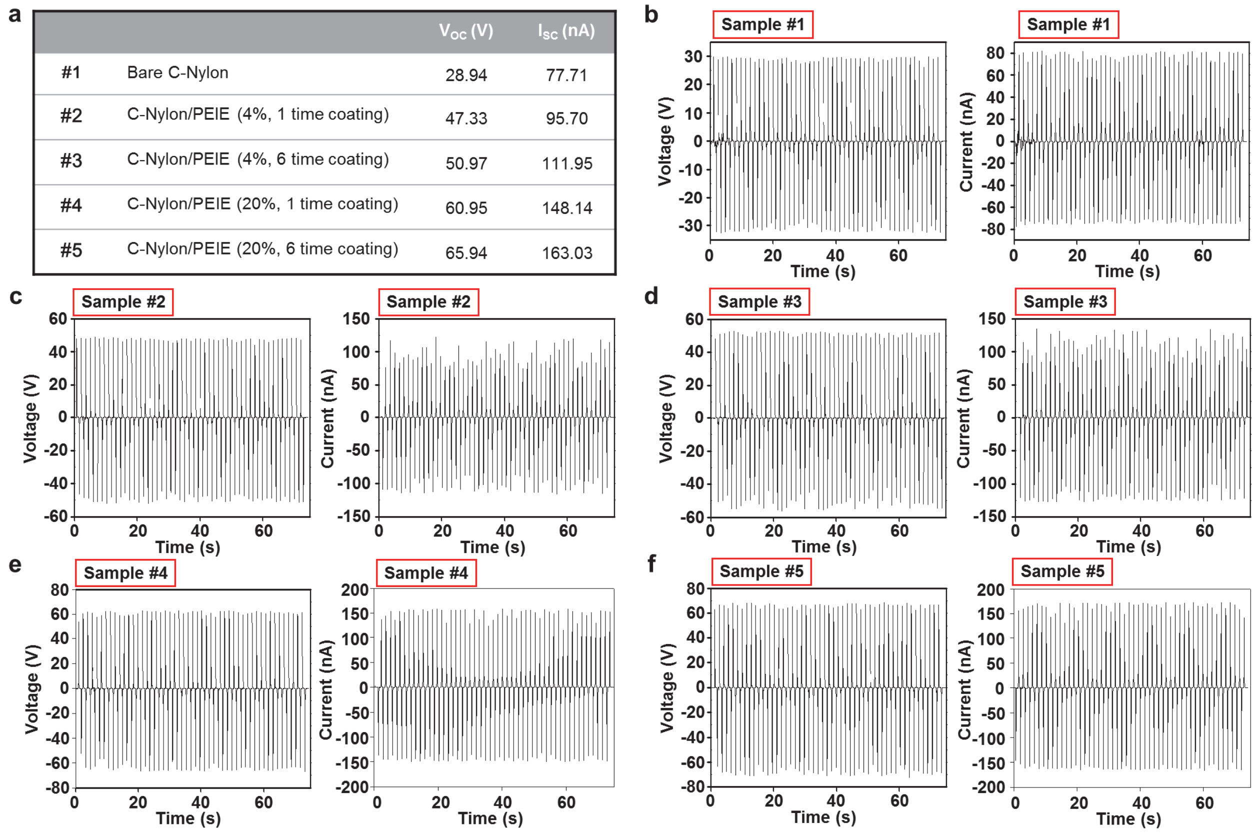

		V_{OC} (V)	I_{SC} (nA)
#1	Bare C-Nylon	28.94	77.71
#2	C-Nylon/PEIE (4%, 1 time coating)	47.33	95.70
#3	C-Nylon/PEIE (4%, 6 time coating)	50.97	111.95
#4	C-Nylon/PEIE (20%, 1 time coating)	60.95	148.14
#5	C-Nylon/PEIE (20%, 6 time coating)	65.94	163.03

Figure S5 | Effects of surface modification. **(a)** Conditions of sample preparation and performance of power generation. Open-circuit voltage (left) and short-circuit current (right) of **(b)** sample #1, **(c)** sample #2, **(d)** sample #3, **(e)** sample #4 and **(f)** sample #5.

Supplementary 6:

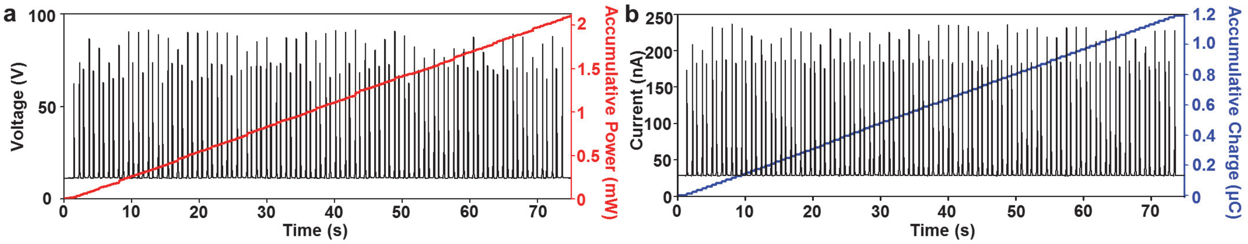

Figure S6 | (a) Rectified voltage (black) and accumulative power (red), **(b)** current (black) and accumulative charge (blue) generated by the sample of optimized features (Ecoflex thickness: 2 mm, PEIE modification: five times using 20 % solution).

Supplementary 7:

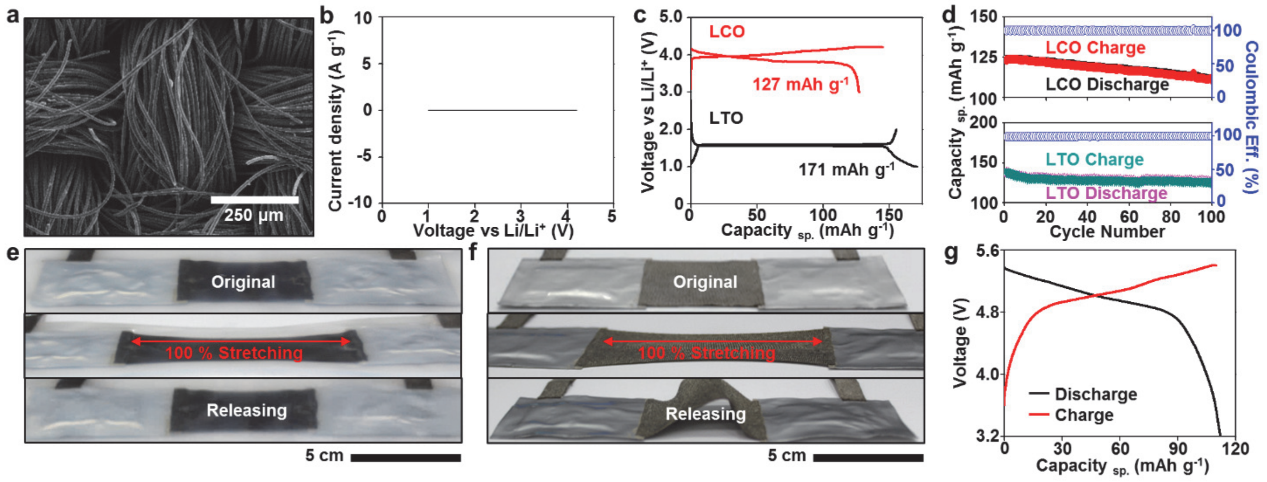

Figure S7 | (a) SEM image of carbon fabric utilized as a current collector in this work. **(b)** Cyclic voltammetry curve of carbon fabric current collector in a voltage range of 1.0–4.2 V (vs. Li/Li^+). **(c)** Voltage profiles, and **(d)** cycle performances and coulombic efficiencies of LCO and LTO half cells. Comparison between the mechanical stability of C-Nylon interconnect **(e)** with/ **(f)** without Ecoflex encapsulation after 100 % of stretching and releasing. **(g)** Voltage profile of the wristband-type LIB connected in series. A nominal voltage of the wristband-type LIB is twice than that of a single pouch cell.

Supplementary 8:

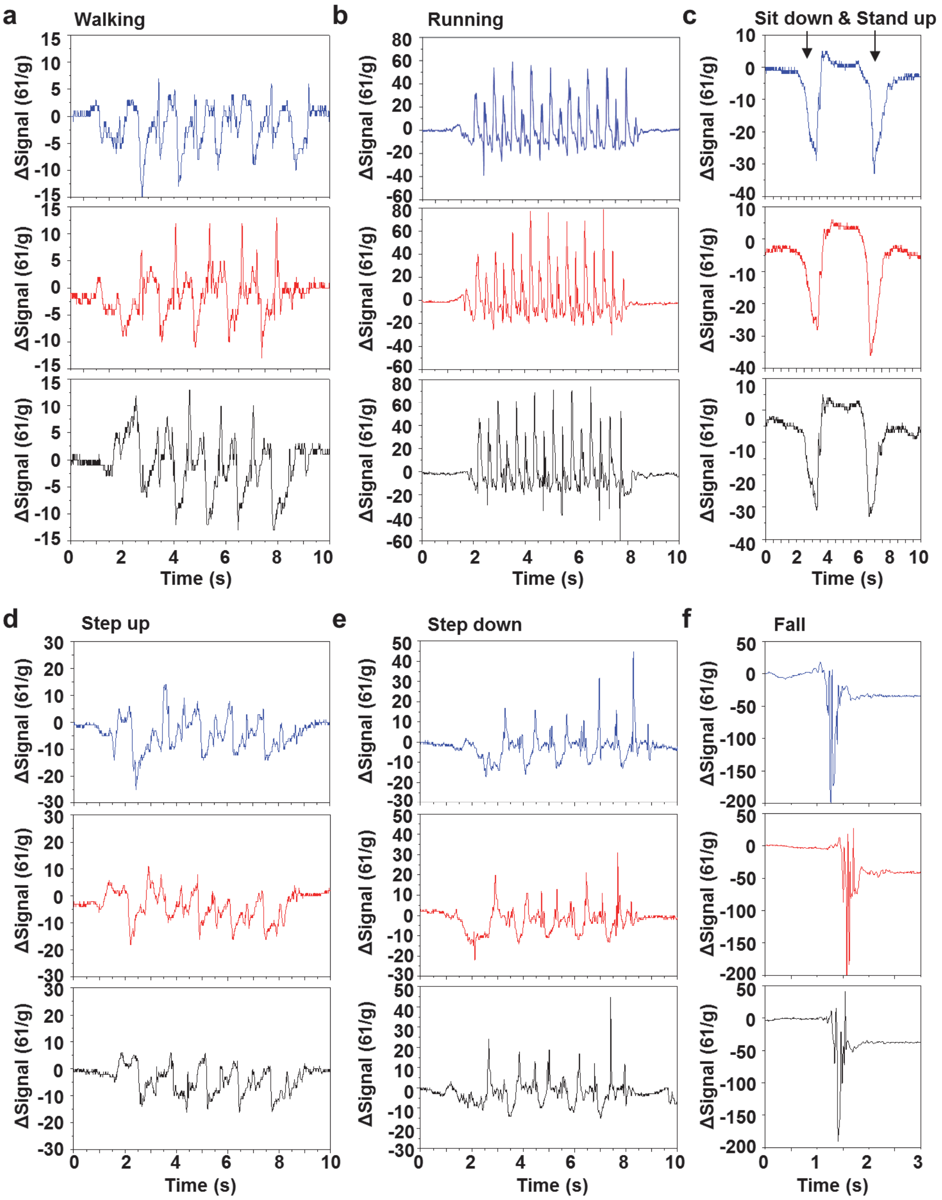

Figure S8 | Vector sums obtained by three different experiments (blue, red, and black curves) for **(a)** walking, **(b)** running, **(c)** sitting up/down, **(d)** stepping up stairs, **(e)** stepping down stairs, and **(f)** falling.

Supplementary 9:

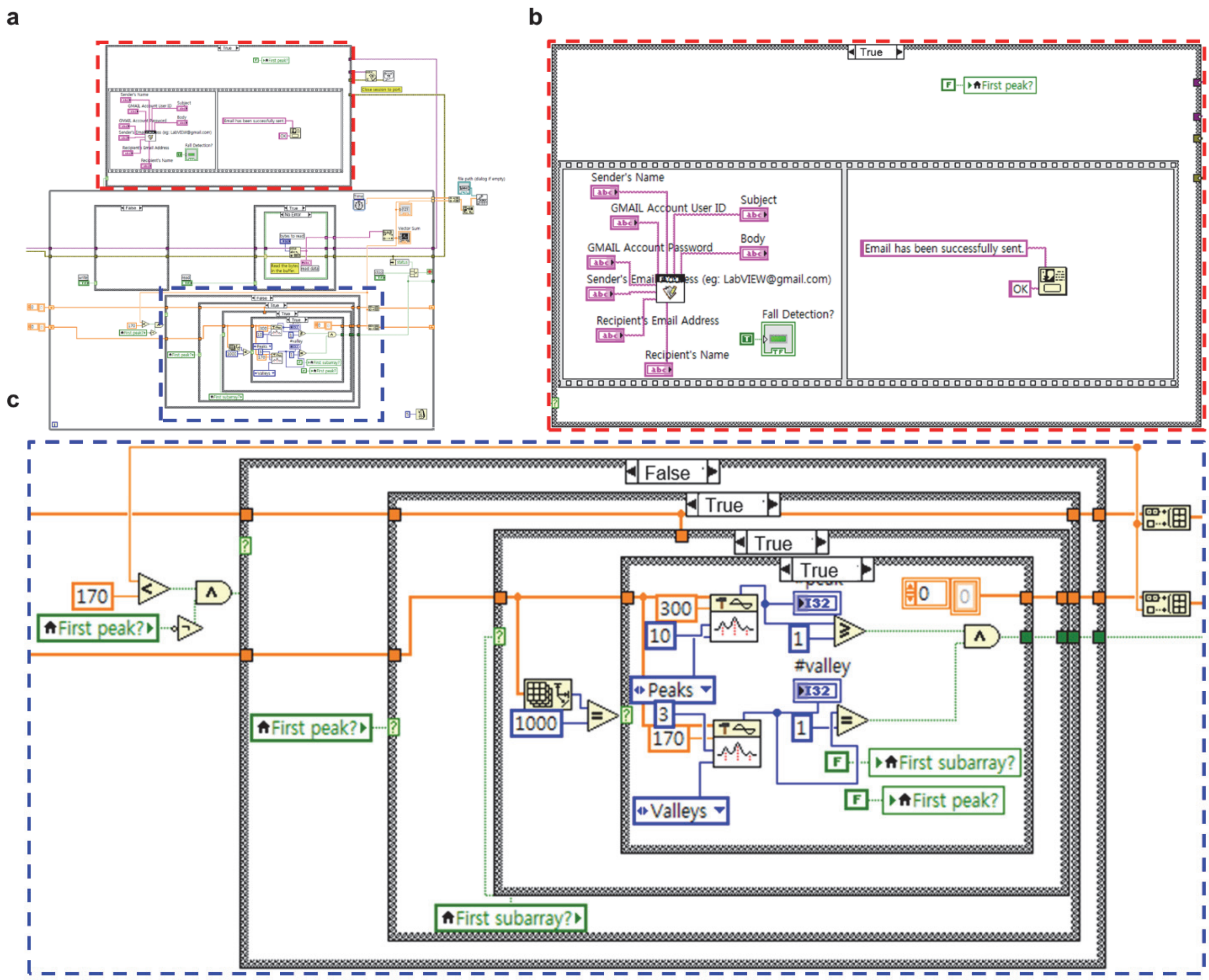

Figure S9 | (a) Part of the software code including the algorithm for sending email (red dotted box) and detecting a fall (blue dotted box). **(b)** Enlarged view of the code for sending email. **(c)** Enlarged view of the code for detecting a fall.

Supplementary Video 1:

Video S1 | Demonstration of integrated wearable fall detector for monitoring simulated motions during daily life.

Supplementary References

1. Forward, K. M. research summary, http://www.csupomona.edu/~kmforward/research.html, accessed: May, 2015.
2. Keyence Corporation, mechanism for the generation of static electricity, http://www.keyence.com/ss/products/static/resource/feature/index.jsp, accessed: May, 2015.
3. Matsusaka, S., Maruyama, H., Matsuyama, T. & Ghadiri, M. Triboelectric charging of powders: A review. *Chem. Eng. Sci.* **65**, 5781-5807 (2010).
4. Wang, Z. L. Triboelectric Nanogenerators as New Energy Technology for Self-Powered Systems and as Active Mechanical and Chemical Sensors. *ACS Nano* **7**, 9533-9557 (2013).
